# Support for mechanical advantage hypothesis of grasping cannot be explained only by task mechanics

**DOI:** 10.1038/s41598-022-14014-2

**Published:** 2022-06-17

**Authors:** Banuvathy Rajakumar, Swarnab Dutta, S. K. M. Varadhan

**Affiliations:** Department of Applied Mechanics, Indian Institute of Technology Madras, Tamil Nadu, Chennai, 600036 India

**Keywords:** Motor control, Neurophysiology

## Abstract

Successful object interaction during daily living involves maintaining the grasped object in static equilibrium by properly arranging the fingertip contact forces. According to the mechanical advantage hypothesis of grasping, during torque production tasks, fingers with longer moment arms would produce greater normal force than those with shorter moment arms. Previous studies have probed this hypothesis by investigating the force contributions of individual fingers through systematic variations (or perturbations) of the properties of the grasped handle. In the current study, we examined the validity of this hypothesis in a paradigm wherein the thumb tangential force was constrained to a minimal constant magnitude. This was achieved by placing the thumb on a freely movable slider platform. The total mass of the handle was systematically varied by adding external loads directly below the center of mass of the handle. Our findings suggest that the mechanical advantage hypothesis manifests only during the heaviest loading condition when a threshold difficulty is reached. We infer that the support for the mechanical advantage hypothesis depends not only on the physical parameters but also on the individual ability to manage the task.

## Introduction

Among all the activities of daily living, grasping and manipulating objects is one of the most crucial and fundamental actions performed by humans. Maintaining the grasped object in static equilibrium is perhaps a critical aspect that governs safe handling by avoiding clumsy behavior such as dropping and spilling. Any form of perturbations to the grasped object requires a complete rearrangement of the fingertip forces to sustain the static equilibrium.

Previously, studies have been performed to investigate the force contribution of the individual fingers and thumb when systematic variations (or perturbations) were imparted to the properties of the grasped handle. Such variations involved introducing external torques^1–4^, varying the mass of the external loads^5,6^, surface friction modification^7–9^, alteration to the grip width of the handle^10^ or individual digit width^11^ in the horizontal direction, and change in the position of the fingertips^12^ and the thumb^3^ while grasping. Normal and tangential forces of the individual fingers and thumb varied systematically in response to these perturbations. Some of these perturbations also disturbed the rotational equilibrium of the handle. In such situations, compensatory torque production was required by the fingers to sustain the handle in static equilibrium. Considering the position of the thumb as pivot point (located midway between middle and ring finger) while grasping a handle, peripheral fingers (index and little) have longer moment arm for normal force than the central fingers (middle and ring) with shorter moment arm for the normal force. According to the mechanical advantage hypothesis (MAH)^13,14^, during compensatory moment production tasks, for example, when there is a requirement to produce supination moment (or torque) in the clockwise direction, little finger with longer moment arm for normal force tends to produce greater normal force than its neighboring ring finger with shorter moment arm for normal force. Thus, by utilizing the mechanical advantage of the little finger, the total force produced by the ulnar fingers could be reduced without compromising on the required moment^15^.

Several studies have attempted to examine the validity of the principle of mechanical advantage for a five-finger grasping task. Mechanical advantage hypothesis was supported in tasks that involved handle rotation in the pronation and supination directions at two different speeds^16^, the addition of an external load of different masses at varying distances from the center of mass of the handle^17^, and moment production to follow a trapezoid template by pressing with all four fingers^18^. However, the hypothesis was only partially supported in a moment production task on a mechanically fixed object^19^, where the distance from the fingers to the axis of rotation, magnitude, and direction of torque production was varied systematically. The authors of the afore-mentioned study posited that the applicability of the MAH may be task and effector-specific. As such, it is yet unclear what kind of tasks the applicability of MAH depends on. Therefore, it is necessary to investigate whether the applicability of mechanical advantage is task-specific and which kind of tasks/scenarios support the MAH.

In our previous study^20^, we had attempted to investigate the applicability of MAH by introducing torque changes to the handle. Rather than implementing external torque changes by suspending the load at a distance from the center of mass of the handle, we incorporated the torque changes by reducing friction between the thumb platform and the handle interface. This was made possible by placing the thumb on a slider platform that could freely translate vertically over a railing. In this way, the tangential force produced by the thumb was kept constant and less than the virtual finger^21^ (an imaginary finger whose mechanical output is equal to the combined output of the individual fingers except for the thumb). This had resulted in introducing a residual pronation torque to the handle. Since the instruction was to maintain the handle in static equilibrium, a compensatory supination torque was required to avoid the tilt caused as a result of the residual pronation torque. Ulnar finger normal forces and thumb tangential forces are major contributors to this compensatory supination torque. However, by our design, it was not possible to increase the tangential force of the thumb as it had to hold the slider platform steady at the HOME position (midway between middle and ring fingers). Therefore, only the normal forces produced by the ulnar fingers became the primary source of this compensatory supination torque.

Between the ulnar fingers, the little finger has a larger moment arm for normal force when compared with the ring finger. Hence it was expected that the little finger would produce greater normal force. Contrary to this expectation, ring and little fingers were found to share comparable normal forces while grasping the handle of mass 0.535kg^20^. Therefore, in the current study, we expected that MAH would be corroborated if the mass of the handle is increased systematically by adding different external loads. As per the design of this grip device, the tangential force of the thumb was constrained to a constant minimal magnitude. So, with an increase in the mass of the handle, only the tangential force of the virtual finger increases, which is accompanied by an increase in the residual pronation torque. As a corrective effect, the magnitude of compensatory supination torque required to be produced would also increase. Hence, we expected that with a systematic increase in the mass of the handle, the little finger would produce correspondingly higher normal force than the ring finger during compensatory supination torque production.

In line with such an expectation, in the current study, the mass of the handle was systematically increased by employing external loads of mass 0.150, 0.250, 0.350, and 0.450 kg as different experimental conditions. Our previous study had shown comparable normal forces between the ulnar fingers for a handle mass of 0.535kg^20^. In another study on investigating the role of grasp force magnitude during multi-finger prehension^22^, by suspending an external load of mass 0.160 kg eccentrically at various distances under a handle of mass 0.415 kg, the contribution of digit forces in terms of percentage of total normal force of the virtual finger was examined. It was found that even for a small external torque of 0.14 Nm, during natural grasping, the percentage share of the little finger normal force (approx. 45%) was greater than the ring finger normal force (approx. 33%).

The total mass of the handle (0.450 kg) with the minimum external load (0.150 kg) used in our current study was approximately close to the total mass of the grip device in the afore-mentioned multi-finger prehension study^22^ in which MAH was supported. Hence, we hypothesized that the mechanical advantage hypothesis would be supported for all our experimental conditions (0.150, 0.250, 0.350, and 0.450 kg) of external load starting with a minimal mass of 0.150 kg (Hypothesis H1).

## Methods and materials

### Participants

Twelve young, healthy right-handed male volunteers participated in this study. The mean and standard deviation of the participant’s age, height, weight, hand length, and width were measured as follows: Age: 26.75 ± 3.9 years, Height: 172.02 ± 5.7 cm, Weight: 75.21 ± 17.7 kg, Hand-length: 18.93 ± 1.1 cm, and Hand-width: 8.92 ± 0.7 cm). Only participants with no history of neurological diseases and musculoskeletal injuries were chosen to participate in this experiment.

### Ethics approval

The Institutional Ethics committee of the Indian Institute of Technology Madras approved the experimental procedures (Approval Number: IEC/2021-01/SKM/02/05). All the participants gave written informed consent according to the procedure approved by the institutional ethics committee of IIT Madras before the beginning of the experiment. The experimental sessions were conducted by strictly adhering to the procedures approved by the Institutional Ethics Committee of the Indian Institute of Technology Madras.

### Experimental setup

**Figure 1:**
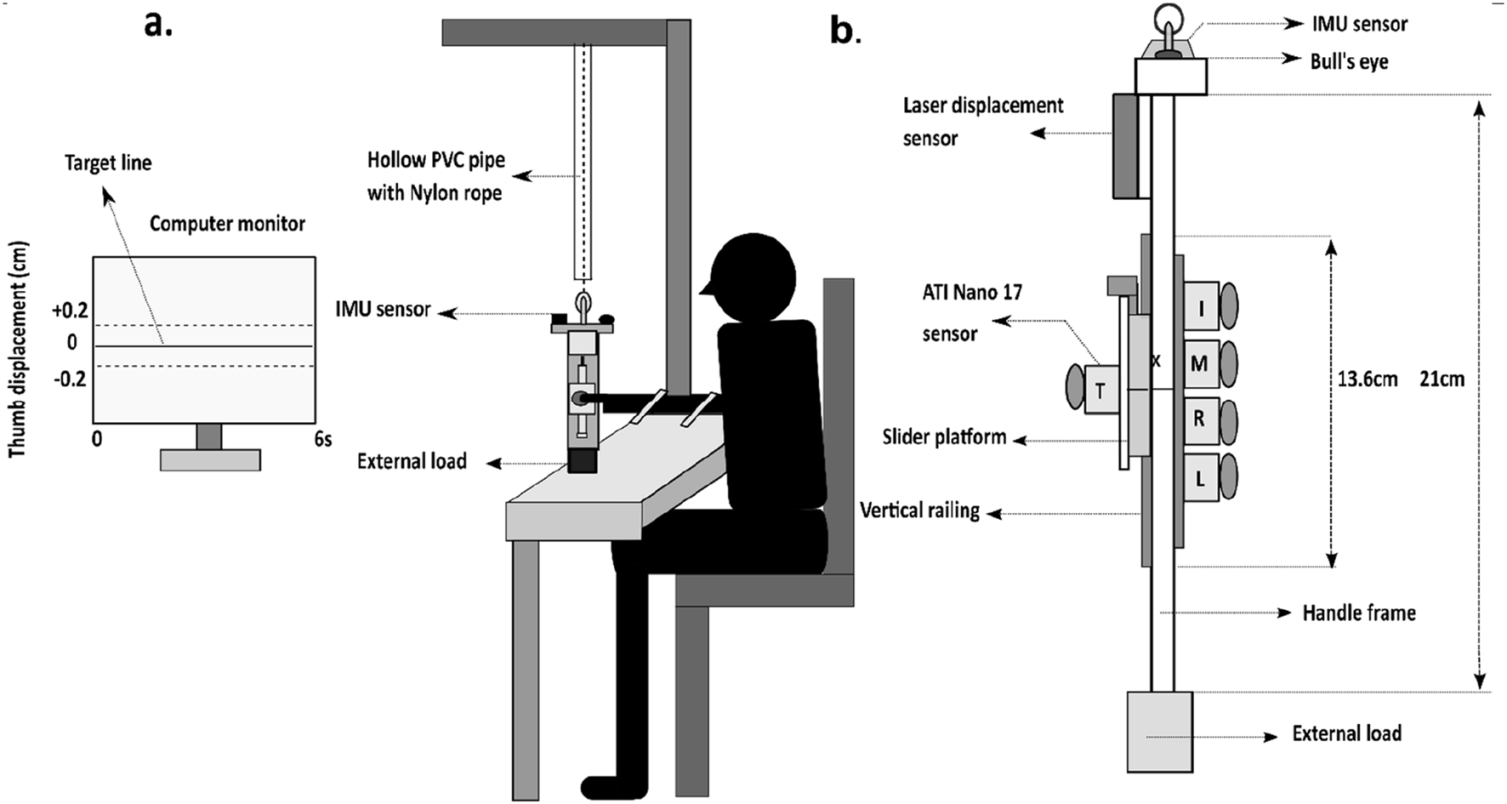
Schematic diagram of the experimental setup and five-finger prehensile handle. (**a**) Experimental setup with the participant holding the handle at a distance of 1.5 m away from the computer monitor. The handle was suspended from a wooden support using a nylon rope housed within the hollow PVC pipe to restrict the lateral movements of the handle. The solid horizontal target line was shown on the computer monitor with two dashed lines that represented an acceptable error margin. (**b**) Schematic diagram of the experimental handle. The aluminium handle frame (21 × 1 × 3) cm with five fingertip force (ATI Nano 17) sensors, laser displacement sensor, and orientation measuring sensor (IMU) are shown. The grip aperture of the handle is 6.2 cm. External loads of 0.150, 0.250, 0.350, and 0.450 kg were attached at the bottom of the handle (i.e.) below the center of mass (represented as ‘X’) of the handle. I, M, R, L, T represents Index, Middle, Ring, Little, and Thumb.

A five-finger prehensile handle was designed and custom-built for the experiment, as shown in Fig. 1 (refer Supplementary Video S6). The handle consists of a vertical railing of length 13.6 cm fitted on the thumb side to mount the slider platform, thus allowing its vertical translation along the railing. The handle was suspended from wooden support using a nylon rope housed within a hollow PVC pipe to restrict any undesirable lateral movement while it was suspended. The present study involves a prismatic precision grip of the handle of mass 0.450 kg. The mass of the slider platform was 0.100 kg. Thus, restricting the thumb tangential force to approximately 1 N. Five six-axis force/torque sensors (Nano 17, Force resolution: Tangential: 0.0125 N, Normal: 0.0125 N, ATI Industrial Automation, NC, USA) was mounted on the handle to measure the forces and the moments exerted by the individual fingers and thumb. For the thumb alone, the force sensor was placed on the slider platform, which enabled the smooth translation of the platform over the railing fitted on the handle's thumb side.

A laser displacement sensor (resolution, 5 μm; OADM 12U6460, Baumer, India) was mounted on a square flat piece made of acrylic, and the assembly was fitted on top of the handle towards the thumb side. The displacement sensor provided the displacement data of the thumb platform in the vertical direction while it translated along the vertical railing. On top of the handle, another acrylic block was placed in the anterior–posterior direction, which held an intelligent 9-axis absolute orientation sensor (Resolution: 16bits, Range: 2000°/s, Model: BNO055, BOSCH, Germany). This IMU (Inertial Measurement Unit) sensor provided the orientation data of the handle after appropriate pre-processing of the raw data. A spirit level was also mounted on the acrylic block towards the participant’s side of the handle to aid the participant in ensuring the handle’s vertical orientation while it was being held.

Two horizontal lines were drawn on the participant’s side of the handle, one at the center of the thumb platform and another midway between middle and ring fingers on the handle frame. The participants were asked to hold the handle in a way such that the two lines were aligned. Thirty analog signals from the force/torque sensors (5 sensors × 6 components) and single-channel analog laser displacement data were digitized using NI USB 6225 and 6002 at 16-bit resolution (National Instruments, Austin, TX, USA). This data was synchronized with four channels of processed, digital data from the IMU sensor. Sampling rates of all data were set to 100 Hz.

### Experimental procedure

Participants were asked to wash and clean their hands with soap, towel-dry and then sit comfortably on a wooden chair with their forearm resting on the table. The right upper arm was abducted at approximately 45° in the frontal plane, flexed 45° in the sagittal plane, and elbow flexed at approximately 90°. The natural grasping position can be achieved by supinating the forearm at 90°. The movements of the forearm and wrist were constrained by fastening with a Velcro strap to the tabletop.

The experiment involved four conditions. For these conditions, external loads of mass 0.150, 0.250, 0.350, and 0.450 kg were added at the bottom of the handle, i.e., exactly under the center of mass of the handle. A computer monitor displayed a solid horizontal target line with two dashed lines at 0.2 cm above and below the target line. These dashed lines represented an acceptable error margin. The target line shown on the monitor corresponded to the ‘HOME position’ of the thumb. The trial began only after the participant could hold the thumb platform steadily by aligning the horizontal line on the thumb platform to the line drawn midway between the middle and ring finger. Thumb displacement data measured using a laser displacement sensor was shown as feedback on the participant’s screen. Once the trial started, the participants were required to keep the slider platform in the same position (HOME), by aligning the horizontal line on the platform to the line drawn between the middle and the ring fingers. Precise alignment of the two lines during the task essentially meant that the feedback line traced the actual target line. Acceptable performance or task success during the trial was defined to be within an error margin of ± 0.2 cm as mentioned above. Throughout the trial, the handle had to be maintained in static equilibrium in the frontal plane for all the external loads. This was ensured by having the bubble of the spirit level at the center throughout the trial.

For each experimental condition, 25 trials were performed. Each trial lasted for six seconds. One minute of break was provided between trials. After every twelve trials, ten minutes of break was provided to eliminate the effect of over exertion, if any. The experiment was held in two separate sessions. Each session included two external load conditions with thirty minutes of break between conditions. The order of presentation of these two sessions was counterbalanced across all participants (see Supplementary Table S7). Six of the participants performed with the weight of 0.150 kg followed by 0.350 kg in their first session. The other six participants performed with the weight of 0.450 kg followed by 0.250 kg in their first session (refer Supplementary Note and Fig. S8).

### Data analysis

The data were analyzed offline using MATLAB (Version R2016b, MathWorks, USA). Force/Torque data and laser displacement data of thumb were lowpass filtered at 15 Hz using second-order, zero phase lag Butterworth filter. In each trial, the data between 2 and 5 s were taken for analysis to avoid start and end effects.

The normal and tangential force data collected from the individual fingertips and the thumb were averaged over the time samples, trials, and participants for each condition separately, and the standard errors of the mean were computed.

### Statistics

All Statistical analyses were performed using R. Two-way repeated-measures ANOVA was performed on the average normal force with the two factors being *loads* (4 levels: 0.150, 0.250, 0.350, 0.450 kg) and *fingers* (4 levels: index, middle, ring, little). Since the thumb normal force is dependent on the normal forces of the index, middle, ring, and little fingers, a separate one-way repeated measures ANOVA was performed on the thumb normal force with the factor as *loads* (4 levels: 0.150, 0.250, 0.350, 0.450 kg). Another two-way repeated-measures ANOVA was performed on the average tangential force with the factors being *loads* (4 levels: 0.150, 0.250, 0.350, 0.450 kg) and *fingers* (5 levels: index, middle, ring, little, thumb). Sphericity test was done on the data, and the number of degrees of freedom was adjusted by Huynh–Feldt (H–F) criterion wherever required. Pairwise post hoc tukey tests were performed to examine the significance within factors. Further, we performed equivalence tests for all the non-different pairs. The statistical equivalence was tested using the two one-sided t-tests (TOST) approach^23^ for a desired statistical power of 95%. The smallest effect size of interest (SESOI) was chosen as the equivalence bounds.

## Results

### Task performance

All the participants were able to trace the horizontal target line shown on the monitor within the error margin during all four loading conditions (0.150, 0.250, 0.350, and 0.450 kg) as shown in Supplementary Fig. S1. Root mean squared error (RMSE) on the thumb displacement data was computed for the four different loads and is shown in Table 1. Throughout the trial, the participants attempted to maintain the handle in static equilibrium during all four loading conditions by positioning the bubble at the center of the bull’s eye. Therefore, the average net tilt angles for the different loading conditions were found to be less than one degree, as shown in Table 1. Thus, the participants could trace the target line with minimal vertical displacement and minimal tilt during all trials in all loading conditions.

**Table 1:** Root Mean Square Error on the thumb displacement data and Net tilt angle.

Additional loads (kg)	Net tilt angle (degrees) (mean ± SD)	RMSE on the thumb displacement data (cm)
0.150	0.58 ± 0.22	0.0215 ± 0.0054
0.250	0.73 ± 0.19	0.0246 ± 0.0066
0.350	0.70 ± 0.15	0.0240 ± 0.0071
0.450	0.81 ± 0.23	0.0325 ± 0.0185

The table shows the average net tilt angle measured in degrees and root mean square error (RMSE) in cm on the thumb displacement data with standard deviation for the four different loads 0.150, 0.250, 0.350, and 0.450 kg.

### Normal forces of the individual fingers and thumb during different loads

The normal forces of the ring and little fingers were found to be statistically comparable with the addition of external loads of 0.150, 0.250, and 0.350 kg. However, when an external load of 0.450 kg was added, the little finger normal force was found to be statistically (*p* < 0.0001) greater than the ring finger normal force and thus supporting MAH.

**Figure 2:**
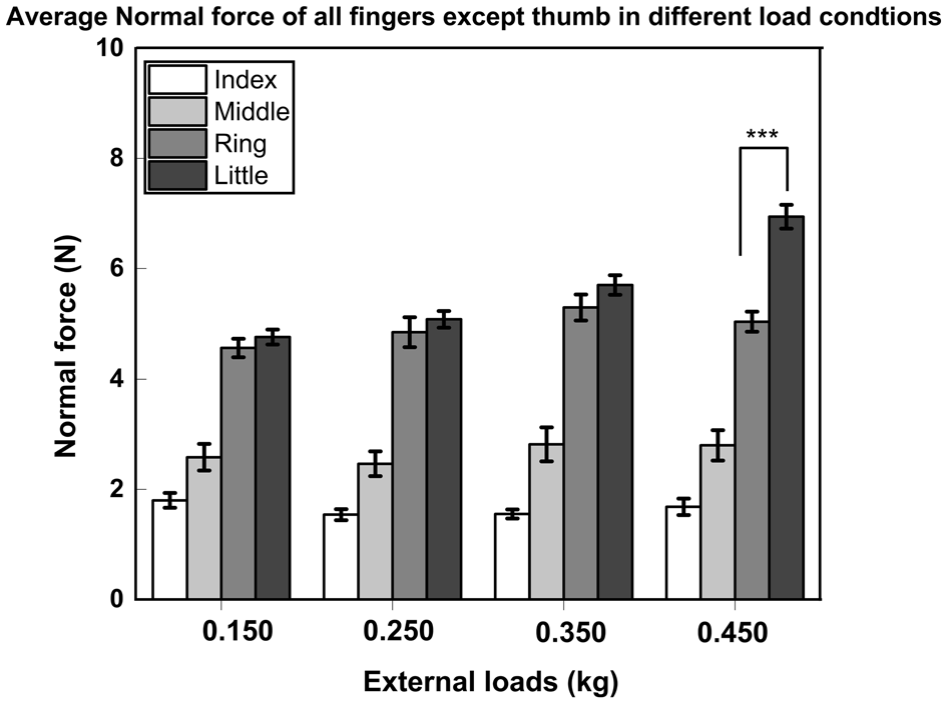
Average Normal force of Index, Middle, Ring, and Little fingers under different loading conditions. Little finger normal force (represented in black) was found to be statistically (*p* < 0.0001) greater than the ring finger normal force (represented in dark shaded grey) in the 0.450 kg loading condition. The ring and little finger normal forces were found to be statistically equivalent under remaining loading conditions. The columns and bars indicate means and standard errors of means. (Note: *** represents significance of less than 0.0001).

We observed a main effect of the factor *loads* (F_(2.73, 30.03)_ = 8.571; *p* < 0.001, η^2^_p_ = 0.43) when a two-way repeated-measures ANOVA was performed on the absolute normal force with the factors as *loads* and *fingers*. It was found that the normal forces of the individual fingers (excluding the thumb) under the loading condition of 0.450 kg were statistically (*p* < 0.001) greater than the normal forces produced under loading conditions of 0.150 and 0.250 kg. Further, the normal forces produced with a load of 0.350 kg were statistically (*p* < 0.05) greater than the normal force produced with a load of 0.150 kg. In addition to this, there was a significant effect of the *fingers* (F_(3, 33)_ = 181.921; *p* < 0.001, η^2^_p_ = 0.94) corresponding to a statistically (*p* < 0.001) higher normal force by the little finger than the index, middle and ring fingers on loading (refer Fig. 2). Also, the normal force of the ring finger was statistically greater than the index and middle fingers.

Meanwhile, we also found that the ring and little finger normal forces were non-different when external loads of masses 0.150 kg (t(11) = − 1.129, *p* = 0.283, d_z_ = 0.32), 0.250 kg (t(11) = − 0.978, *p* = 0.349, d_z_ = 0.28) and 0.350 kg (t(11) = − 1.454, p = 0.174, d_z_ = 0.41) were employed. Therefore, by using TOST procedure on the dependent pairs (0.150 kg: Ring: Mean = 4.55 N, SD = 0.55; Little: Mean = 4.75 N, SD = 0.45; 0.250 kg: Ring: Mean = 4.84 N, SD = 0.90; Little: Mean = 5.07 N, SD = 0.50; 0.350 kg: Ring: Mean = 5.29 N, SD = 0.78; Little: Mean = 5.70 N, SD = 0.58), it was confirmed that the ring and little finger normal forces were statistically equivalent (0.150 kg: t(11) = 2.473, *p* = 0.0155; 0.250 kg: t(11) = 2.625, *p* = 0.0118; 0.350 kg: t(11) = 2.148, *p* = 0.0274) with the observed effect size that falls within the equivalence bounds of ∆_L_ = − 1.04 and ∆_U_ = 1.04 under the loads of 0.150, 0.250 and 0.350 kg.

**Figure 3:**
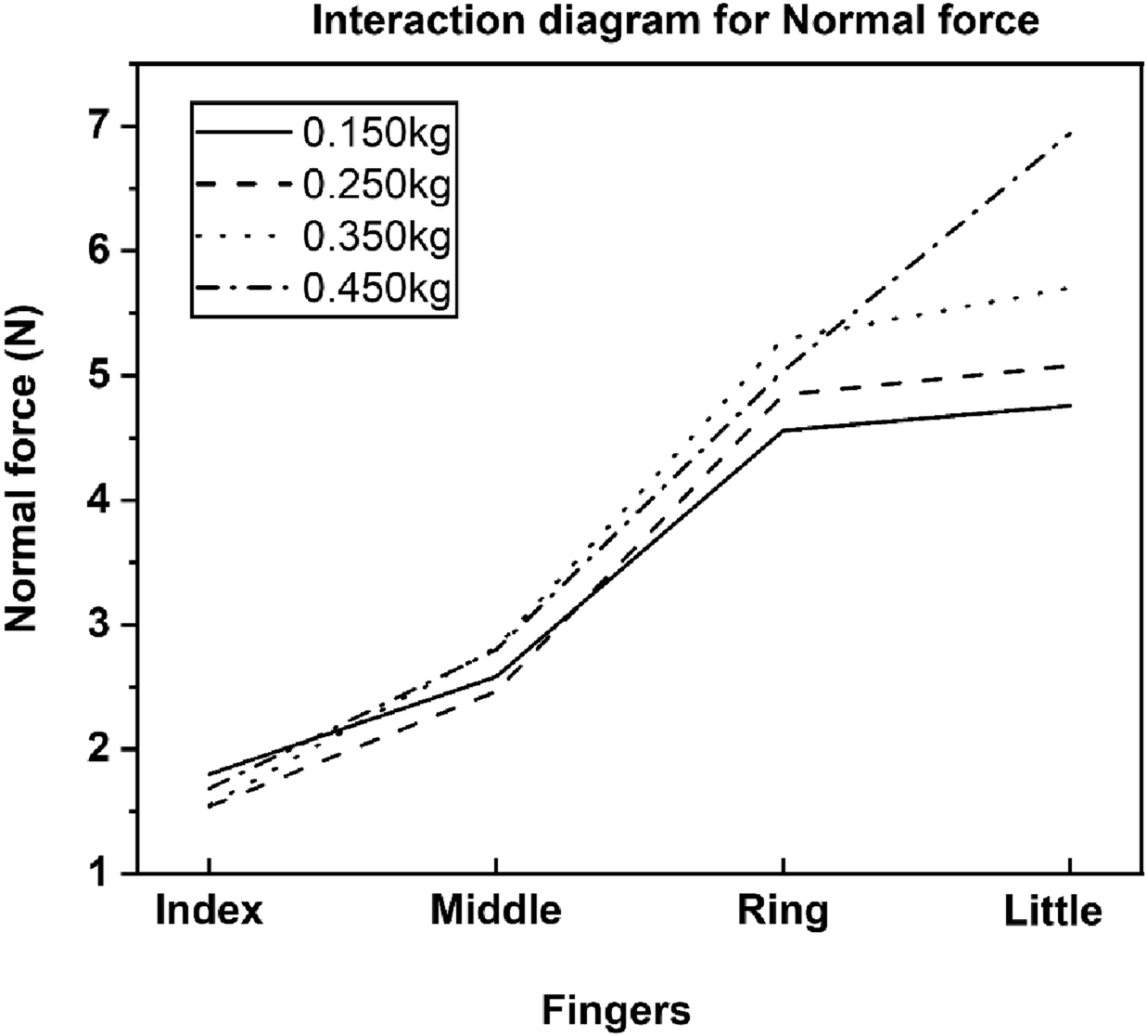
Interaction between loads and finger normal forces The pairwise post hoc tukey tests confirmed that the ring and little finger normal forces of 0.450 kg (Ring: 5.03 N, Little: 6.94 N) 0.350 kg (Ring: 5.29 N, Little: 5.70 N), 0.250 kg (Ring: 4.84 N; Little: 5.07 N) were statistically greater than the index and middle finger normal forces (0.150 kg: Index: 1.79 N, Middle: 2.58 N; 0.250 kg: Index: 1.54 N, Middle: 2.46 N; 0.350 kg: Index: 1.55 N, Middle: 2.81 N; 0.450 kg: Index: 1.68 N, Middle: 2.79 N) of all the loading conditions.

The interaction *loads* × *fingers* was significant (F_(3.96, 43.56)_ = 18.538; *p* < 0.001, η^2^_p_ = 0.62) reflecting the fact that the ring and little finger normal forces of 0.450 kg (Ring: 5.03 N, Little: 6.94 N), 0.350 kg (Ring: 5.29 N, Little: 5.70 N), 0.250 kg (Ring: 4.84 N; Little: 5.07 N), were statistically (*p* < 0.001) greater than the index and middle finger normal forces (0.150 kg: Index: 1.79 N, Middle: 2.58 N; 0.250 kg: Index: 1.54 N, Middle: 2.46 N; 0.350 kg: Index: 1.55 N, Middle: 2.81 N; 0.450 kg: Index: 1.68 N, Middle: 2.79 N) of all the loading conditions (refer Fig. 3).

While, the pairwise post hoc tukey tests confirmed that the little finger normal force (6.94 N) of 0.450 kg was statistically (*p* < 0.001) greater than the ring and little finger normal forces (0.150 kg: Ring: 4.55 N, Little: 4.75 N; 0.250 kg: Ring: 4.84 N, Little: 5.07 N; 0.350 kg: Ring: 5.29 N, Little: 5.70 N (*p* < 0.01)) of the remaining loads (see Fig. 3). Whereas a little finger (5.70 N) of 0.350 kg produced statistically (p < 0.01) greater normal force than the ring finger (4.55 N) under 0.150 kg of load.

One-way repeated-measures ANOVA was performed on the thumb normal force, which showed a significant effect of the factor loads (F_(2.69, 88.90)_ = 9.411; *p* < 0.001, η^2^_p_ = 0.46). Under the loading of 0.450 kg, the thumb normal force (16.50 N) was found to be statistically greater than under loadings of 0.150 kg (13.73 N, *p* < 0.01) and 0.250 kg (13.97 N, *p* < 0.05) (see Supplementary Fig. S2).

### Tangential forces of individual fingers during different loads

In the case of the tangential forces, a two-way repeated-measures ANOVA with the factors as *loads* (F_(3, 33)_ = 390.575; *p* < 0.001, η^2^_p_ = 0.97) and *fingers* (F_(4, 44)_ = 44.205; *p* < 0.001, η^2^_p_ = 0.80) showed significant effect of the factor *loads* corresponding to a statistically greater tangential force with the use of 0.450 kg than with the use of 0.150 kg (*p* < 0.001), 0.250 kg (*p* < 0.001) and 0.350 kg (*p* < 0.05). In addition, a significant effect of the factor *fingers* confirmed that the little finger tangential force was statistically (*p* < 0.001) greater than the index, middle, and ring finger tangential forces on loading.

**Figure 4:**
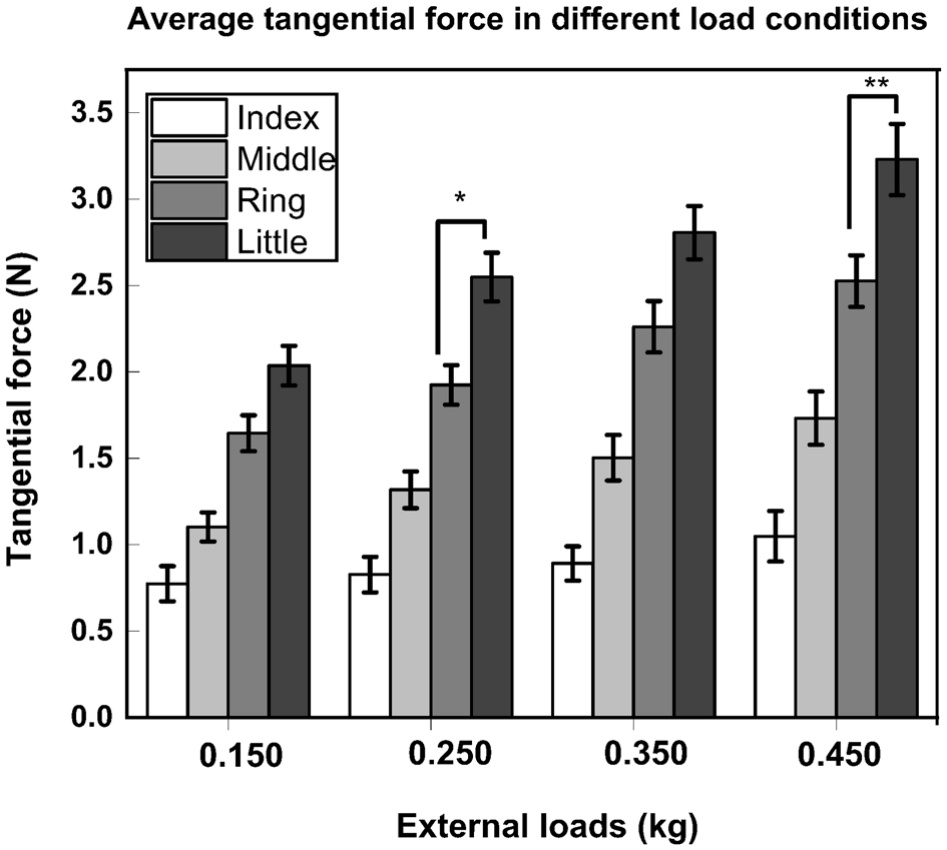
Average tangential force of Index, Middle, Ring, and Little under different loading conditions. Little finger tangential force (0.250 kg: 2.54 N; 0.450 kg: 3.22 N) was found to be statistically greater than the ring finger tangential force (0.250 kg: 1.92 N; 0.450 kg: 2.52 N) under 0.250 kg (*p* < 0.05) and 0.450 kg (*p* < 0.01) loading conditions. In particular, the little finger tangential forces (3.22 N) of 0.450 kg was statistically greater than the little finger tangential forces (0.150 kg: Little: 2.03 N; 0.250 kg: Little: 2.54 N) of 0.150 and 0.250 kg. The columns and bars indicate the means and standard errors of means. (Note: ** represents significance of less than 0.01 and * represents significance of less than 0.05).

In addition to this, on performing the pairwise post hoc tukey test, it was confirmed that the little finger tangential force (0.150 kg: 2.03 N, 0.350 kg: 2.80 N) was non-different from the ring finger tangential force during the employment of 0.150 kg (1.64 N) and 0.350 kg (2.26 N). TOST procedure performed on these dependent pairs confirmed that the comparisons were not statistically equivalent. However, little finger tangential force (0.450 kg: 3.22 N; 0.250 kg: 2.54 N) was statistically greater than the ring finger tangential force with the addition of load 0.450 kg (Ring: 2.52 N, *p* < 0.01) and 0.250 kg (Ring: 1.92 N, *p* < 0.05) (refer Fig. 4).

Further, interaction effect of *loads* x *fingers* was significant (F_(12, 132)_ = 5.857; *p* < 0.001, η^2^_p_ = 0.34) reflecting the fact that the little finger tangential forces (3.22 N) due to the use of 0.450 kg was statistically greater than the ring and little finger tangential forces (0.150 kg: Ring: 1.64 N, *p* < 0.01, Little: 2.03 N, *p* < 0.001; 0.250 kg: Ring:1.92 N, *p* < 0.001, Little: 2.54 N, *p* < 0.05; 0.350 kg: Ring: 2.26 N, *p* < 0.001) due to the other loads (refer Supplementary Fig. S3).

## Discussion

The main objective of the present study was to investigate whether the applicability of MAH is dependent solely on task parameters like the total weight of the handle, moment arm of the suspended load, or is it affected by factors beyond these physical parameters. We tested the manifestation of MAH in our paradigm by systematically increasing the weight of the handle by adding external loads at the bottom of the handle below its center of mass. The weight of our current handle with the minimal loading condition exceeded the weight of the handle in our previous study^20^. So, we hypothesized that MAH would be supported in all our loading conditions. Contrary to our expectation, we found that the ring and little finger normal forces were statistically comparable with the addition of 0.150, 0.250, and 0.350 kg loads. However, we noticed that MAH was supported for the external load of 0.450 kg. We discuss the implications of these findings in the following paragraphs.

Ulnar finger normal forces were examined under four different external loading conditions i.e., 0.150, 0.250, 0.350 and 0.450 kg. In our previous study, with a similar unsteady thumb platform as used in the current study, the ulnar fingers produced comparable normal forces for a handle mass of 0.535 kg^20^. With the minimal external load of 0.150 kg, the total mass of the handle would become 0.600 kg (above 0.535 kg that was used in our previous study). So, our expectation was that MAH would be supported for all the loading conditions. In contrast to our expectation, the little finger produced statistically comparable normal forces to the ring finger for 0.150 kg load. With further increase in the external loadings with masses 0.250 and 0.350 kg, the ulnar fingers continued exhibiting statistically comparable normal forces. However, this trend did not hold true when the external load was increased to 0.450 kg, wherein the little finger exerted a statistically greater normal force than the ring finger.

Unlike the other studies on grasping with eccentrically loaded manipulanda, the current study involved maintaining a constant minimal tangential force by the thumb (approximately 1 N) at different loading conditions (see Supplementary Fig. S4). Therefore, with an increase in the total mass of the handle by adding an external load of 0.450 kg (comparatively larger than the mass of other loads employed in the present study), the virtual finger had to share greater tangential force to maintain the vertical equilibrium causing a greater pronation torque (counter-clockwise direction from the participants viewpoint). This, in turn, necessitated progressively greater compensatory supination moments to maintain the rotational equilibrium. Since the design of the handle prevents the thumb from contributing further to the supination moment, the ulnar fingers are required to compensate with their normal forces. In this regard, instead of exerting comparable normal forces, the little finger tends to produce greater normal force than the ring finger, thus supporting MAH. What could be the reason for this behavior of ulnar fingers with the addition of the heaviest external load as compared to the other loads?

The next natural question is whether the applicability of mechanical advantage depends on employing heavy masses while grasping. If that had been true, then MAH would have been supported when a large external load of 2 kg was suspended at a distance of 1.9 cm from the center of mass (COM) of the handle (for a torque magnitude of − 0.375Nm) in the grasping study investigating the contribution of peripheral and central fingers^17^. However, they found that ulnar fingers normal forces were non-different for this large load. Eventually, with a systematic increase in the compensatory supination torque magnitude (0.750, 1.125, and 1.50Nm), the little finger gradually started producing more normal force than the ring finger and validated the MAH.

In another study investigating the role of grasp force magnitude during multi-finger prehension^22^, when an external load of mass 0.160 kg (much lesser than 2 kg) was suspended from a handle of mass 0.415 kg eccentrically at a distance of 8.9 cm from COM, MAH was supported. This result triggers another question as to whether the support for MAH depends on suspending the external load at large moment arms from COM of the handle? From the results of the multi-finger prehension study^22^, it was apparent that the applicability of mechanical advantage depends on using higher moment arms for the external load. Our current result forces us to re-evaluate this conclusion, as MAH was supported even when an external load of 0.450 kg was suspended directly below COM of the handle (having zero moment arm). This suggests that apart from the mass of the external load and moment arm of the suspended load, a latent factor governs the applicability of mechanical advantage. In other words, our data suggest that the applicability of the principle of mechanical advantage in biological systems depends not only on the mass or moment arm of the suspended load or both but also on more individual-specific components such as the individual ability of managing a task.

In the prehension study investigating the effect of grasp force magnitude^22^, the demanding aspect of the task might have been using an unusually high moment arm, thus allowing MAH to manifest. In a recent study^24^ using a handle similar to the current study, the task was to trace trapezoid and inverted trapezoid patterns by displacing the thumb platform 1.5 cm above and below the HOME position. The mechanical advantage hypothesis was supported during the inverted trapezoid condition when the movable thumb platform was held steady while tracing the static portion 1.5 cm below the HOME (at the level below the center of the ring finger sensor). The carpometacarpal joint (CMC) of the thumb has a restricted range of motion in the downward direction^25^ (flexion or radial adduction). Therefore, the task of maintaining the handle in static equilibrium with a movable thumb platform at the level below the center of the ring finger sensor might have been quite difficult to perform. We suggest that this biomechanical constraint which imparted difficulty in accomplishing the task may have caused the little finger to share greater normal force than the ring finger.

Following a similar rationale, in the current study, perhaps the task became fairly demanding, as the requirement was to produce compensatory supination torque with only the normal forces of the ulnar fingers. This was a direct effect of restricting the thumb tangential force to a constant minimal magnitude and essentially rendering it much less consequential in the supination torque production. Simultaneously, this also amplified the role of the ulnar fingers in the compensatory torque production. For the heaviest external load of 0.450 kg, the magnitude of fingertip forces required were much higher than the magnitude of fingertip forces in the relatively easier loading conditions i.e., for the 0.150, 0.250, 0.350 kg loads. According to a study that investigated the use of mechanical advantage in multi-finger torque production^15^, MAH is employed to reduce the total effort or force produced for the task without compromising to produce the required moment. Along similar lines, we speculated that to avoid higher exertion (higher force levels) of the ulnar fingers by sharing comparable and greater forces (due to tangential force restriction in the thumb), the participants used the mechanical advantage of the little finger to more efficiently manage the grasp after a threshold difficulty was reached. As per the instruction to participants in the current study, the participants were allowed to continue performing the trials only when they did not feel over exertion or pain. Therefore, to successfully complete the task, without indulging in straining the ring and little fingers, participants would have chosen to minimize the total force (or effort) in the ulnar fingers by employing the principle of mechanical advantage.

Also, from literature^26^, it was found that the exclusion of the little finger from the overall grip pattern decreased overall grip strength by 33%, and exclusion of the ring finger from the overall grip pattern decreased overall grip strength by 21%. This shows that, among the ring and little fingers, little finger contribution is fairly higher than the ring finger when there is an increase in overall grip force requirement. Thus, an addition of heavier external load, which in turn increases overall grip force requirement, might have caused the little finger to contribute significantly greater than the ring finger. From an anatomical perspective, the little finger has an additional group of intrinsic muscles (hypothenar muscles) compared to the ring finger, which could be a supporting factor to employ little finger than ring finger when the task becomes difficult or demanding.

To further elucidate our result, it is important to emphasize that in the previous studies on object manipulation that introduced external torques to the handle, there was no restriction in the distribution of tangential forces among the fingers and the thumb while grasping. The tangential force of the thumb would have greatly contributed to the supination torque in addition to the normal forces of the ulnar fingers. This was evident from the previous study^17^, where the thumb tangential force increased during the supination efforts. Hence, the participants might have been able to share comparable normal forces by the ring and little fingers even with a larger load (2 kg) and with a greater torque magnitude of 0.375 Nm than in the current study.

In contrast, in our current study, the tangential force of the thumb was restricted to approximately 1 N by placing the thumb on a freely movable platform of mass 0.100 kg for all the loading conditions. This essentially creates a situation wherein the ulnar fingers are forced to contribute greatly to the compensatory supination torque. We posit that such a constraint in the tangential force contribution of the thumb is most exemplified under 0.450 kg external load. Note that this load is much less than the 2 kg load where MAH was not supported. We strongly believe that individual-specific components such as the individual ability of managing a task that is difficult to accomplish might have encouraged the use of the mechanical advantage of the little finger. The difficulty faced by the performer is not dictated merely by the external loads and torques but also due to the biomechanical constraint as in the previous study^24^, or it could be due to the individual’s ability of managing a task.

**Figure 5:**
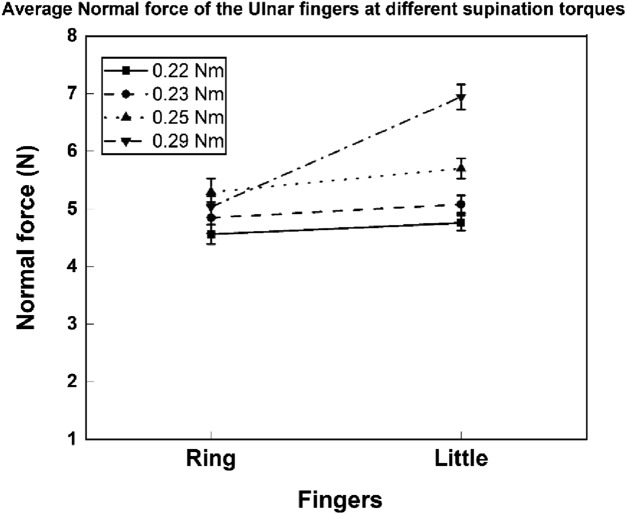
Normal forces of ring and little fingers for supination efforts under different loading conditions/supination torque requirement. For 0.29 Nm torque requirement (represented by dashes with dots), the normal force of little finger was found to be statistically greater than ring finger normal force. In all the other conditions, ring and little fingers produced comparable normal force. The bars indicate standard errors of means.

In the present study, the participants could complete the task under loads of 0.150, 0.250, and 0.350 kg (resulting in the supination torques of 0.22, 0.23, and 0.25 Nm, respectively), which might not have been difficult enough than with a load of mass 0.450 kg (refer Fig. 5 and Supplementary Fig. S5). As under a load of mass 0.450 kg, the task of maintaining the static equilibrium of the handle by producing greater and comparable forces by the ulnar fingers might have been difficult. Therefore, for successful completion of the task, little finger having both mechanical and anatomical advantage would have produced greater force than ring finger. Whereas, due to the task simplicity, comparable normal forces would be produced by the ulnar fingers, with the addition of 0.150, 0.250, and 0.350 kg loads.

In a study on producing maximum voluntary contraction MVC^27^, when the target finger is the little finger, the force produced by the little finger was found to be well above the adjacent ring finger force. Analogously, since our study involved very strong voluntary grasping of the handle for the external loading condition of 0.450 kg, greater activation of the little finger motor units could have caused a greater force in the little finger than the ring finger, thus enabling optimal distribution of forces within the ulnar fingers in line with the MAH. This is also supported by the study^28^ wherein they found that the magnitude of force produced due to the little finger motor units under the ring finger was almost two-thirds of the force produced under little finger during voluntary grasping. Since we have not measured the actual activation pattern of the individual motor units, further research is required to tease out the underlying neural mechanisms through which mechanical advantage is manifested. Taken together, our results suggest that the applicability of the mechanical advantage hypothesis depends not only on the torque requirement or the total mass of the object but also on the individual’s ability to manage the task.

## Concluding comments

The current study was performed to validate whether the mechanical advantage hypothesis is task-specific and investigate the kind of tasks that lend support to the MAH. A five-finger prehensile handle with an unsteady thumb platform was utilized for analysing the applicability of MAH. The mass of the handle was systematically increased by using additional external loads of mass 0.150, 0.250, 0.350, and 0.450 kg. Ulnar fingers exerted comparable normal forces with the external loads of mass 0.150, 0.250, and 0.350 kg. However, the mechanical advantage hypothesis was supported with a load of 0.450 kg. With the addition of greater mass, under the constraint of using minimal thumb tangential force, establishing static equilibrium by the ulnar fingers becomes progressively more challenging. Therefore, we conclude that MAH as a strategy utilised in human grasping is not only employed when there is any change in the mass of the grasped handle or moment arm of the suspended load but also when a certain threshold difficulty is reached during the task.

## Supplementary Information

Supplementary Information 1.

Supplementary Video 1.

## Data Availability

We plan to publish a data descriptor article along with this manuscript. Hence the data will be made available in due course of time. The data descriptor article is now under consideration at Scientific Data.
