# Supplementary material for: Support for mechanical advantage hypothesis of grasping cannot be explained only by task mechanics
Source: Sci Rep. 2022 Jun 17;12:10242. doi: 10.1038/s41598-022-14014-2 (PMC9206022; doi:10.1038/s41598-022-14014-2)
Supplement: Supplementary file 1 — Supplementary Information 1. [file 41598_2022_14014_MOESM1_ESM.docx]

**Support for mechanical advantage hypothesis of grasping cannot be explained only by task mechanics**

**Banuvathy Rajakumar^1^, Swarnab Dutta^2,^** and **Varadhan SKM^3*^**

^1,2,3^Department of Applied Mechanics, Indian Institute of Technology Madras, Chennai, India

**Corresponding Author:**

Dr. Varadhan SKM

Associate professor

Department of Applied Mechanics

Indian Institute of Technology Madras

Chennai - 600036

Tamil Nadu

+91-44-22574071

Email address: skm@iitm.ac.in

Corresponding Author ORCID: 0000-0002-5746-2340

| 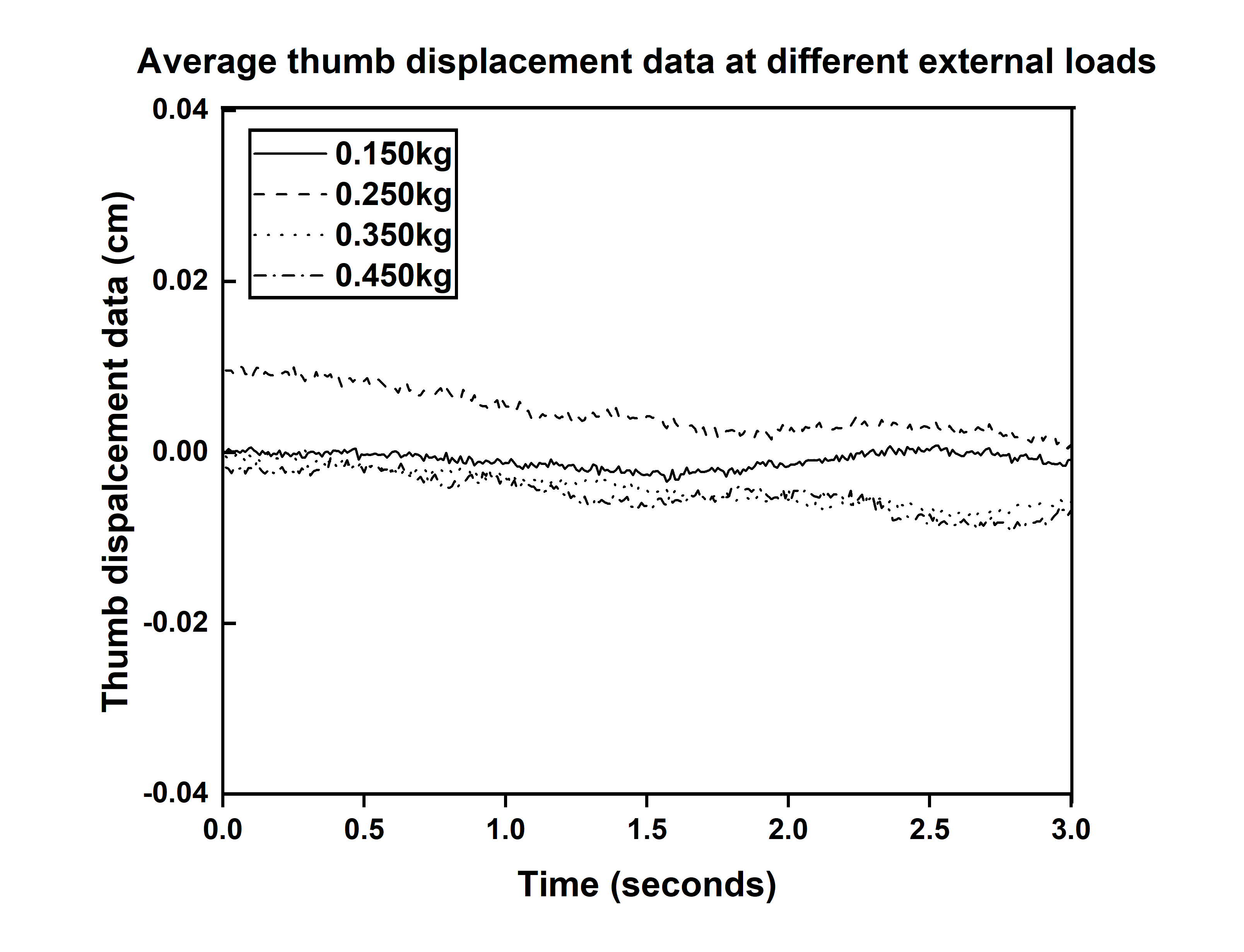 |
| --- |
| **Supplementary Figure S1 Average thumb displacement data at different load conditions.** Each line plot represents the average taken across trials and participants during the addition of each external load. The solid horizontal target line displayed on the computer monitor appears at 0cm (not shown in this figure for the sake of clarity). X axis represents the time in seconds. Since we consider only the data from 2s to 5s for analysis, only the 3s data (sampling frequency =100Hz) is shown here. The average thumb displacement data collected in each condition was found to remain closer to the 0cm (i.e target line). |

**Supplementary Information file**

|  |
| --- |
| **Supplementary Figure S2 Average normal force of the thumb under different loading conditions** The thumb normal force (16.50N) with an addition of external load of **0.450kg** was found to be statistically greater than the thumb normal force under the loadings of **0.150kg** (13.73N) and **0.250kg** (13.97N). Further, the thumb normal force (16.50N) at **0.450kg** load was statistically equivalent to the thumb normal force (15.44N) with the load of **0.350kg**. |

| 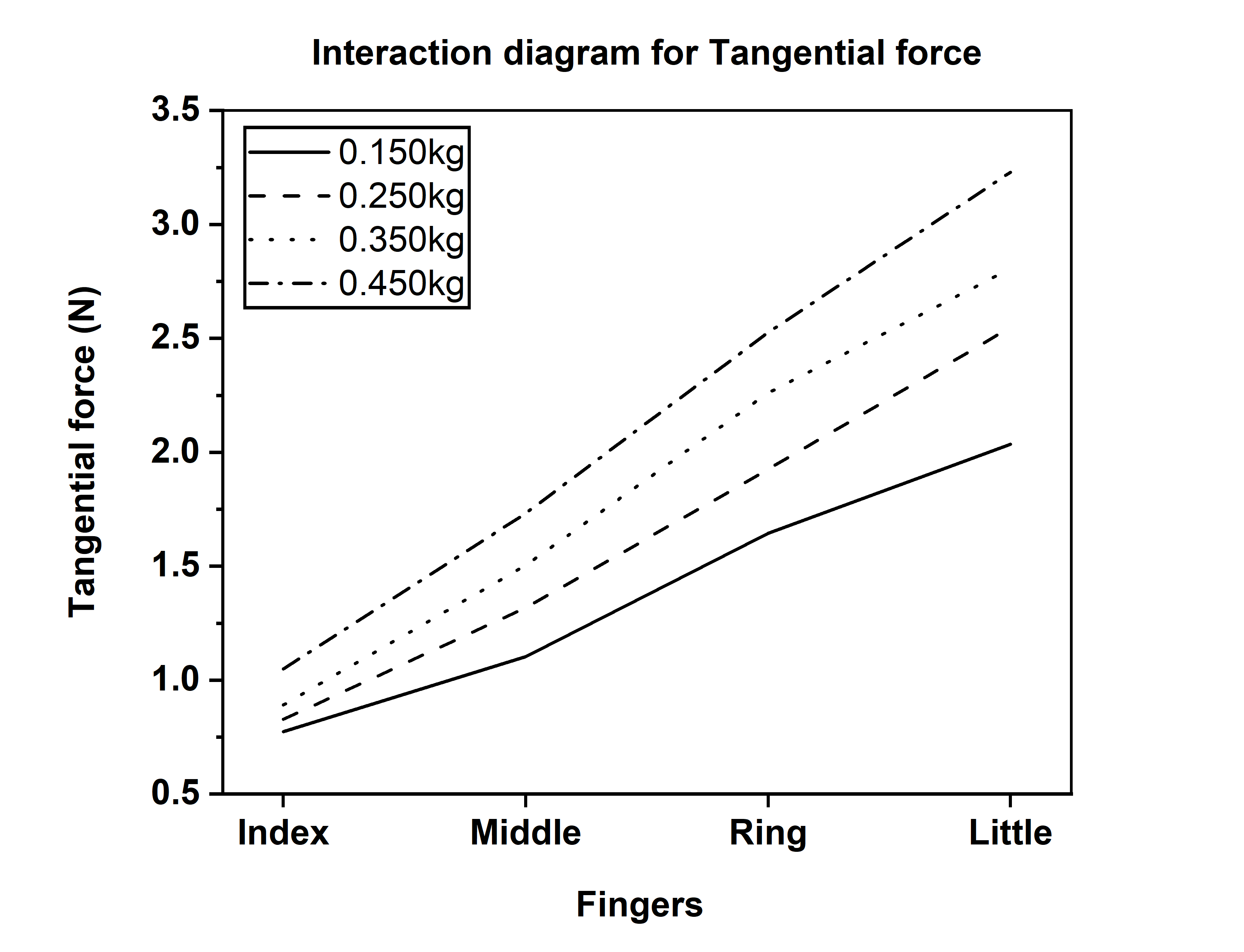 |
| --- |
| **Supplementary Figure S3 Interaction between loads and finger tangential forces** The little finger tangential force (3.22N) with the use of external load of **0.450kg** was statistically greater than the ulnar fingers tangential forces (**0.150kg**: Ring: 1.64N, Little: 2.03N; **0.250kg**: Ring:1.92N, Little: 2.54N) under the loadings of **0.150kg & 0.250kg.** Also, the little finger tangential force (3.22N) at **0.450kg** was statistically greater than the ring finger tangential force (2.26N) when a load of **0.350kg** was added. |

|  |
| --- |
| **Supplementary Figure S4 Average tangential force of Thumb under different loading conditions** Thumb tangential force at different conditions were found to be statistically comparable. |

|  |
| --- |
| **Supplementary Figure S5 Relationship between individual finger normal force and finger for supination efforts under different loading conditions/supination torque requirement.** The results of the pairwise post hoc Tukey tests confirmed that the little finger normal force (6.94N) due to the addition of **0.450kg** load was statistically greater than the ring (**0.150kg**: 4.55N; **0.250kg**: 4.84N; **0.350kg**: 5.29N; **0.450kg**: 5.03N) finger normal force due to the addition of all four different loads. Further, the little finger normal force with the use of **0.450kg** was found to be statistically greater than the little finger (**0.150kg:** 4.75N; **0.250kg:** 5.07N; **0.350kg**: 5.70N) normal forces due to addition of other loads. |

**Supplementary Video S6. Video of a participant performing a trial.** The interface shown in the video is slightly different where all lines are shown as **solid**. While, during the actual experiment, the interface shown to the participants consists of a solid horizontal target line with two dashed lines at 0.2cm above and below the target line. The visual feedback line of the thumb position is shown in ‘white’. The video was shot by the author Banuvathy Rajakumar and the participant is the other author Swarnab Dutta and both give permission for the video to be uploaded.

**Supplementary Note**

0.150kg was performed first in the session 1 of the participant-1 which was then followed by 0.350kg. For the same participant, in the second session, the first load of 0.450kg was used which was then followed by the second load of 0.250kg. The table shown below provide the order of loads in all twelve participants.

**Supplementary Table S7.** Order of the conditions of each participant within each session.

|  | **Session 1** | | **Session 2** | |
| --- | --- | --- | --- | --- |
| **Participant** | **First load** | **Second load** | **First load** | **Second load** |
| Participant 1 | **0.150kg** | **0.350kg** | 0.450kg | 0.250kg |
| Participant 2 | 0.450kg | 0.250kg | **0.150kg** | **0.350kg** |
| Participant 3 | **0.150kg** | **0.350kg** | 0.450kg | 0.250kg |
| Participant 4 | 0.450kg | 0.250kg | **0.150kg** | **0.350kg** |
| Participant 5 | **0.150kg** | **0.350kg** | 0.450kg | 0.250kg |
| Participant 6 | 0.450kg | 0.250kg | **0.150kg** | **0.350kg** |
| Participant 7 | **0.150kg** | **0.350kg** | 0.450kg | 0.250kg |
| Participant 8 | 0.450kg | 0.250kg | **0.150kg** | **0.350kg** |
| Participant 9 | **0.150kg** | **0.350kg** | 0.450kg | 0.250kg |
| Participant 10 | 0.450kg | 0.250kg | **0.150kg** | **0.350kg** |
| Participant 11 | **0.150kg** | **0.350kg** | 0.450kg | 0.250kg |
| Participant 12 | 0.450kg | 0.250kg | **0.150kg** | **0.350kg** |

**Between sessions**

- With regard to the order effect between sessions, suppose if we consider checking data of 0.450kg load during both the sessions i.e., six participants performed 0.450kg load in second session (after performing low load conditions (0.150kg & 0.350kg) in their first session), remaining six participants performed 0.450kg load in their first session (before the low load conditions (0.150kg & 0.350kg)). Ring and little finger normal forces of each trial of six participants who performed 0.450kg load in their first session was averaged across time samples (25 trials x 6 participants= 150). Similarly, averaging was done across time for all the trials of the remaining six participants who performed 0.450kg in their second session.
- In the below figure, if there existed two different clusters separately, i.e., first session data plotted separately and second session data plotted separately, it would confirm the existence of order effect between the sessions. In contrast, there was no such clusters formed, which shows there does not exist any order effect between session 1 and session 2

|  |
| --- |
| **Supplementary Figure S8. Ulnar finger normal forces due to the addition of 0.450kg load during two sessions.** Individual participant ulnar fingers normal force data due to the addition of 0.450kg load during session 1 and session 2 are shown in the figure. Force data collected during session 1 is represented by the symbol ‘square’ and session 2 is represented by the symbol ‘triangle’. |

**Within session-between conditions**

We agree to the point that we had not had a session with 0.250kg load followed by 0.450kg. Even if we had performed 0.450kg after the 0.250kg, our results may not have been different. The statistically different result of the ulnar fingers due to 0.450kg load would not change regardless of what happens in the previous condition.
